# Characterization of the bronchodilatory dose response to indacaterol in patients with chronic obstructive pulmonary disease using model-based approaches

**DOI:** 10.1186/1465-9921-12-54

**Published:** 2011-04-26

**Authors:** Didier Renard, Michael Looby, Benjamin Kramer, David Lawrence, David Morris, Donald R Stanski

**Affiliations:** 1 Novartis Pharma AG, Basel, Switzerland; 2 Novartis Pharmaceuticals, East Hanover, NJ, USA; 3 Novartis Horsham Research Centre, Horsham, West Sussex, UK

## Abstract

**Background:**

Indacaterol is a once-daily long-acting inhaled β_2_-agonist indicated for maintenance treatment of moderate-to-severe chronic obstructive pulmonary disease (COPD). The large inter-patient and inter-study variability in forced expiratory volume in 1 second (FEV_1_) with bronchodilators makes determination of optimal doses difficult in conventional dose-ranging studies. We considered alternative methods of analysis.

**Methods:**

We utilized a novel modelling approach to provide a robust analysis of the bronchodilatory dose response to indacaterol. This involved pooled analysis of study-level data to characterize the bronchodilatory dose response, and nonlinear mixed-effects analysis of patient-level data to characterize the impact of baseline covariates.

**Results:**

The study-level analysis pooled summary statistics for each steady-state visit in 11 placebo-controlled studies. These study-level summaries encompassed data from 7476 patients at indacaterol doses of 18.75-600 μg once daily, and showed that doses of 75 μg and above achieved clinically important improvements in predicted trough FEV_1 _response. Indacaterol 75 μg achieved 74% of the maximum effect on trough FEV_1_, and exceeded the midpoint of the 100-140 mL range that represents the minimal clinically important difference (MCID; ≥120 mL vs placebo), with a 90% probability that the mean improvement vs placebo exceeded the MCID. Indacaterol 150 μg achieved 85% of the model-predicted maximum effect on trough FEV_1 _and was numerically superior to all comparators (99.9% probability of exceeding MCID). Indacaterol 300 μg was the lowest dose that achieved the model-predicted maximum trough response.

The patient-level analysis included data from 1835 patients from two dose-ranging studies of indacaterol 18.75-600 μg once daily. This analysis provided a characterization of dose response consistent with the study-level analysis, and demonstrated that disease severity, as captured by baseline FEV_1_, significantly affects the dose response, indicating that patients with more severe COPD require higher doses to achieve optimal bronchodilation.

**Conclusions:**

Comprehensive assessment of the bronchodilatory dose response of indacaterol in COPD patients provided a robust confirmation that 75 μg is the minimum effective dose, and that 150 and 300 μg are expected to provide optimal bronchodilation, particularly in patients with severe disease.

## Introduction

Indacaterol is the first long-acting inhaled β_2_-agonist indicated for once-daily maintenance treatment in patients with moderate-to-severe chronic obstructive pulmonary disease (COPD), and has been approved in more than 40 countries (including throughout the European Union) for use at doses of 150 and 300 μg once daily. The efficacy and safety of indacaterol was evaluated in an extensive Phase III clinical programme in which patients received doses of up to 600 μg once daily for up to 52 weeks [1-4]. In an analysis of data from 801 patients with moderate-to-severe COPD after 2 weeks of treatment (Stage 1 of a Phase II/III study employing an adaptive seamless design), indacaterol 150 μg once daily was identified as the lowest dose that was numerically superior to the active comparators (formoterol twice daily and open label tiotropium once daily) and, along with the next highest dose (300 μg), was selected for further evaluation [5]. This additional evaluation (Stage 2 of the adaptive seamless design study) showed that indacaterol 150 and 300 μg provided statistically significant and clinically relevant improvements in trough forced expiratory volume in 1 second (FEV_1_) vs placebo up to 26 weeks [2]. Although indacaterol 150 and 300 μg had similar effects on trough FEV_1_, the higher dose was associated with incremental benefits in terms of symptomatic relief, such as dyspnoea [2], particularly for patients with more severe COPD. Further, the overall clinical trial programme has indicated that indacaterol had a similar safety and tolerability profile across all of the doses evaluated [1-4,6].

Conventional dose-ranging trials rely on hypothesis testing and use placebo corrected mean responses to compare dose levels and determine the existence of a dose response. If at least one dose achieves a statistically significant difference compared with placebo for an appropriate endpoint (e.g. trough FEV_1 _for evaluation of bronchodilators in COPD), a dose response is established and a target dose can be selected as the smallest dose that differs from placebo and has both a clinically relevant effect and an acceptable safety profile [7]. Several such studies have evaluated dose responses for bronchodilators in patients with COPD [8-12]. In Phase II dose-ranging studies in COPD, indacaterol consistently demonstrated bronchodilator efficacy that was superior to placebo, regardless of the dose tested [13,14].

**Figure 1:**
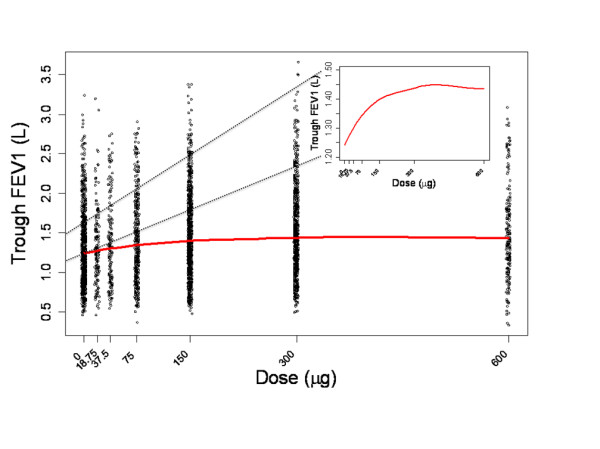
**Individual-patient trough FEV_1 _data with LOESS curve, with zoom-in on the LOESS curve in the range 1200-1500 mL**.

**Figure 2:**
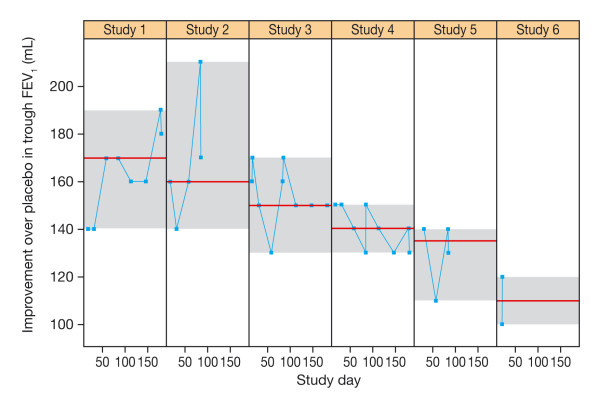
**Improvement in trough FEV_1 _(mL) with indacaterol 150 μg observed at different days in six of the studies in the study-level analysis ranked by median value**.

The potential of indacaterol as a bronchodilator is best appreciated when the responses across all the tested dose levels are expressed together in a dose-response relationship. However, given the inherent variability in measurements of lung function relative to the drug-induced change achieved by bronchodilators, accurate characterization of the dose response relationship is difficult. Figure 1 shows individual-patient trough FEV_1 _data over a range of indacaterol doses (using data from the studies included in the patient-level analysis discussed below) and includes a locally weighted scatterplot smoothing (LOESS) curve to highlight the main trend. While the overall FEV_1 _in the population varied from about 0.5 to 3 L, the maximum drug response vs placebo is under 200 mL as depicted in the figure inset. This is indicative of the low signal-to-noise ratio of the bronchodilatory response in COPD. The impact of this issue on the interpretation of study results is best illustrated by considering the variability of a single dose level within and between trials. Figure 2 depicts the variability in trough FEV_1 _response to indacaterol 150 μg across six different studies. Each panel represents the results from one trial. The data points are the least square means (LSM) for each study visit. The grey area within each panel provides a visual representation of the range of responses observed within each trial. The panels are ranked by the median response observed in each trial. This figure shows that the intra- and inter-study variability in mean trough FEV_1 _may be as high as 50 mL, whereas the inter-study variability in median response may be about 60 mL. The implications of this observation is that relying on single LSM values does not provide adequate precision to easily differentiate between dose levels.

To overcome this inherent difficulty in using conventional methodology to accurately establish dose responses for bronchodilators in COPD, two alternative approaches were explored. The first approach focused on the study level results typically reported for bronchodilator assessment. The aim of this approach was to use study level LSM from COPD studies in the indacaterol development programme to provide estimates of the dose response and of the precision of typical study level data used for the purpose of dose selection. The second approach focused on individual patient data from two studies. The aim of this approach was also to determine the dose response while exploring individual patient characteristics that may affect the drug response and hence dose selection. To our knowledge, this is the first time that these novel modelling methods have been used to characterize dose response in COPD patients receiving a bronchodilator.

## Methods

Two approaches were used: 1) an integrated analysis of study-level data, and 2) an integrated analysis of patient-level data. The objectives of both analyses were to provide a precise quantitative characterization of the dose-response relationship of indacaterol and the responses to comparators used in some trials. The key metrics of interest were: the minimum effective dose (MED), defined as the lowest dose that achieved a median trough FEV_1 _that exceeded the midpoint of the 100-140 mL range considered to represent the minimum clinically important difference (MCID) for FEV_1 _in COPD (i.e., a difference from placebo of ≥120 mL) [15]; the optimal dose, defined as the lowest dose that achieved or exceeded the criteria for the MED and was superior to all active comparators; and the maximum dose defined as the lowest dose with a 95% confidence interval (CI) for the predicted response that includes the expected maximum response. A further objective of the patient-level analysis was to determine patient-level characteristics that influenced the dose response, and so may influence dose selection decisions.

### Data sources

The study-level pooled analysis included data from 7476 patients enrolled in 11 placebo-controlled studies in which indacaterol was administered to patients with COPD at doses of 18.75 to 600 μg once daily (Table 1). The analysis involved placebo-controlled studies that included assessment of trough FEV_1 _and had a duration of at least 14 days. Indacaterol was compared with formoterol in two studies, with salmeterol in four studies and with tiotropium in one study. Note all comparator data was at steady-state and assessed at the same study visits in the respective studies. LSM contrasts to placebo and associated standard errors were collected from individual study reports to create the study-level pooled analysis dataset. The LSM estimates were obtained after various covariate adjustments in the original statistical analyses of each individual study (details of covariate adjustments are included in Additional file 1).

**Table 1:** Studies of indacaterol included in the study-level pooled analysis (all studies) and patient-level analysis (B2335S and B2356)

Design	Patients	Indacaterol dose, μg						Pbo	For	Sal	Tio
						
		18.75	37.5	75	150	300	600				
Cross-over, 14-day	96					144^‡^		72		72	

Parallel-group, 52-week	1732					437	428	432	435		

Parallel-group 26-week	2059			130	420	418	123	425	123		420

Crossover, 14-day	68					66		66		65	

Parallel-group, 12-week	416				211			205			

Parallel-group, 12-week	347*				114	116		117			

Parallel-group, 26-week	563				188	188		187			

Parallel-group, 26-week	1002				333			335		334	

Parallel-group, 12-week	323			163				160			

Parallel-group, 12-week	318			159				159			

Parallel-group, 12-week	552	92	91	94	92			91		92	

	7476	92	91	546	1358	1369	551	2249	558	563	420

All studies were placebo-controlled. Values are numbers of patients (the sum of totals across the columns for indacaterol dose and comparators is greater than the total number of patients randomized due to the inclusion of cross-over studies). *Asian patients; ^‡^73 morning dosing vs 71 evening dosing. Pbo = placebo; For = formoterol 12 μg bid; Sal = salmeterol 50 μg bid; Tio = tiotropium 18 μg qd

To evaluate FEV_1 _at steady state, the analysis pooled study results from Week 2 up to Month 6 of therapy. This timescale was selected as indacaterol is known to have reached both pharmacodynamic and pharmacokinetic steady state by Week 2 [2]. For example, in a study of 1683 patients, improvements in mean trough FEV_1 _with indacaterol 150 and 300 μg vs placebo were similar at Weeks 2, 12 and 26, with no decline over this period [2].

The patient-level analysis evaluated trough FEV_1 _in 1835 patients enrolled in two dose-ranging studies in which indacaterol was delivered using the single-dose dry powder inhaler that is used for the commercially-available product. As one of these studies had a duration of 2 weeks and the other had key dose-ranging data over the same duration [5], the patient-level analysis considered trough FEV_1 _measurements only after 2 weeks of treatment.

### Study-level analysis

The primary objective of the study-level analysis was to characterize the dose-response relationship for indacaterol in patients with COPD. The analysis of steady state trough FEV_1 _was conducted using an E_max _model:

(1) $$E=\frac{E_{\text{max}}\times dose}{ED_{50}\times dose},$$

where i is an index for study and j for study arm, E_max _is the (model-predicted) maximum possible response, and ED_50 _characterizes drug potency and corresponds to the indacaterol dose producing 50% of the maximum effect. The model included between-study (δ_i_) and within-study, between-visit (γ_ij_) variability on E_max _and was analysed using a Bayesian methodology.

As the summary data used in this analysis are contrasts to placebo, the model was constrained to have a null response with placebo (dose = 0). Summary information on formoterol, salmeterol and tiotropium, collected in the studies included in this pooled analysis, was also added (complete model equations are described in Additional file 1). The Bayesian analyses were implemented with Markov chain Monte Carlo methods using WinBUGS software version 1.4.3 [16]. For each analysis the posterior distribution of the structural model parameters and key derived parameters were summarized as mean, median, standard deviation, as well as 2.5th and 97.5th quantiles, which provided 95% CIs for each parameter. Data are presented for six indacaterol doses corresponding to the two doses at which indacaterol is approved in many countries (150 and 300 μg), together with doses equal to double the highest approved dose (i.e. 600 μg), half the lowest approved dose (i.e. 75 μg), and two lower doses (18.5 and 37.5 μg). The responses to the comparators are included for reference.

### Patient-level analysis

For the patient-level analysis, a nonlinear mixed effects (NLME) model was used [17], based on an E_max _dose-response model:

(2) $$\begin{array}{c} y_{ijk}=FOR_{ij}\times \mu_{F}+SAL_{ij}\times \left(\mu_{S}+\gamma_{Sij}\right)+TIO_{ij}\times \mu_{T}\\ +IND_{ij}\times \frac{\left(E_{\text{max}}+\delta_{i}+\gamma_{ij}\right)\times dose_{ij}}{ED_{50}+dose_{ij}}+\varepsilon_{ijk}, \end{array}$$

where i is an index for patient and j for study day (14 or 15), E_0 _is the response to placebo, and E_0i _and E_mi _are random effects to account for inter-patient variation in response. NLME models are often used for the purposes of pooling individual patient data as they allow the differences between patients to be accounted for in an unbiased manner as fixed effects (e.g. patient characteristics such as age and disease status) and random effects (e.g. the remaining random differences that cannot be accounted for by patient characteristics).

The base model included inter-individual variability (E_0i _and E_mi_) to account for within-patient correlation of the observed responses, as well as covariate adjustments (effect of baseline FEV_1 _on E_0 _and E_max_, and effect of reversibility following administration of a short-acting β_2_-agonist on E_max_). A transform-both-sides approach was used, with the logarithm transformation applied to both the response and the model. An additive residual error term was specified after log transformation. The primary goal was to derive an estimate of the dose response for the improvement over placebo in trough FEV_1 _based on individual measurements in each patient.

The patient-level analysis incorporated patient characteristics, such as disease-relevant covariates, and enabled evaluation of consistency between the two different modelling approaches. Model building proceeded with a forward entry procedure relying on the likelihood ratio test. Tested covariates were: baseline FEV_1 _(average of pre-treatment FEV_1 _values), COPD severity (moderate or lower vs severe or worse, based on the classification of severity of COPD defined in the GOLD 2007 guidelines [18]) use of inhaled corticosteroids, smoking status (ex vs current smoker), gender, age (<65 years vs ≥ 65 years), study day, and study. The final model equation is described in full in Additional file 1. NLME modelling was carried out using SAS/STAT software (procedure NLMIXED), version 9.2 of the SAS system for Unix. The first order estimation method was specified.

## Results

### Study-level analysis

**Figure 3:**
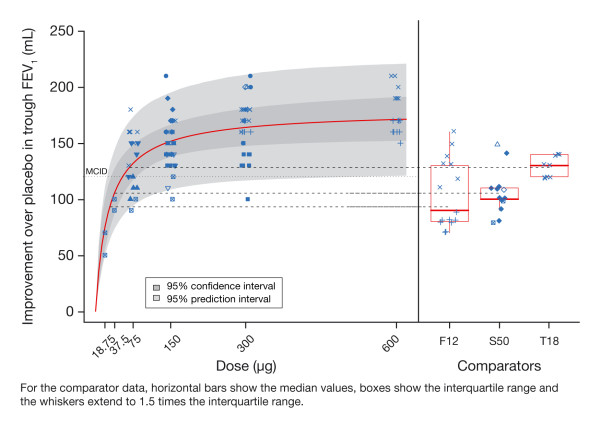
**Prediction of dose response for trough FEV_1 _at steady state in the study-level analysis with comparators**.

The data used in the study-level analysis of trough FEV_1 _are shown in figure 3. Each point represents a LSM contrast to placebo (expressed in mL) as determined for each visit (from Week 2 to the end of the study) and treatment arm of each study, for both indacaterol (left-hand panel) and comparators (right-hand panel). Visual inspection of the indacaterol data points indicated that with increasing dose the response asymptotically approached a maximum plateau. The majority of study results for doses of 75 μg and above exceeded the MCID of 120 mL (dotted line on the graph).

The outcome of the study-level analysis of the 24-h trough values is also presented in figure 3, as the red solid line, representing the mean dose response curve, and two greyed areas representing 95% confidence limits for the curve (darker area) and approximate 95% prediction limits (lighter area) for the data points. The three horizontal dashed lines correspond to mean trough FEV_1 _responses for each of the comparators included in this analysis. The most striking feature of the plot is that the response to indacaterol at the plateau exceeds the response of all comparators. In other words, doses of indacaterol 150 μg or greater provide greater average trough bronchodilation than the comparators.

The mean estimate for the ED_50 _was 28 μg, with a 95% CI ranging between 12 and 52 μg (Table 2); this is the dose that is predicted to produce half the maximum response than can be achieved by indacaterol. The mean E_max _estimate was 177 mL with a 95% CI ranging between 152 and 206 mL; this is the predicted average maximum response. Based on these parameter estimates, the relative potency of the other tested doses can be calculated: 37.5, 75, 150, 300 and 600 μg provided 59, 74, 85, 92 and 96% of the model-predicted maximal effect, respectively (Table 2). This suggests that doses of 75 μg or less are on the steep part of the dose response, 150 μg is at the threshold of the plateau and 300 μg and higher are on the plateau.

**Table 2:** Posterior summaries for parameters from the model of trough FEV_1 _at steady state in the study-level analysis

	Mean	SD	Q2.5	Q50 (median)	Q97.5
Model parameters					

E_max _(mL)	177	13	152	176	206

ED_50 _(μg)	28	10	12	26	52

Derived parameters					

ED_90 _(μg)	110	41	46	105	207

Effect as percentage of maximum effect					

18.75 μg	42	9	27	42	62

37.5 μg	59	9	42	59	76

75 μg	74	7	59	74	87

150 μg	85	5	74	85	93

300 μg	92	3	85	92	96

600 μg	96	2	92	96	98

Q2.5 and Q97.5 are the 2.5th and 97.5th quantiles, respectively, and correspond to the 95% CI for each parameter. SD = standard deviation.

**Figure 4:**
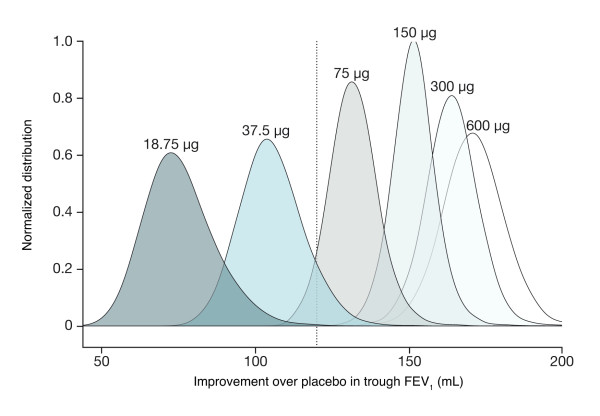
**Posterior distributions of improvement over placebo at steady-state trough FEV_1 _(study-level analysis)**.

A key advantage of a comprehensive quantitative characterization of the dose response is that it allows generation of precise probabilistic statements about the relative responses. Figure 4 presents (normalized) distributions for the mean improvements vs placebo at each dose level that underpin such calculations. Using these distributions, it is possible to calculate, for example, that the probability that the mean improvement vs placebo in trough FEV_1 _for 37.5 μg exceeds the MCID is 7% while the probability that 75 μg exceeds the MCID is about 90% (the corresponding probability was approximately 99.9% for 150 μg). In other words, 75 μg is the most likely lowest tested dose that exceeds the MCID.

**Figure 5:**
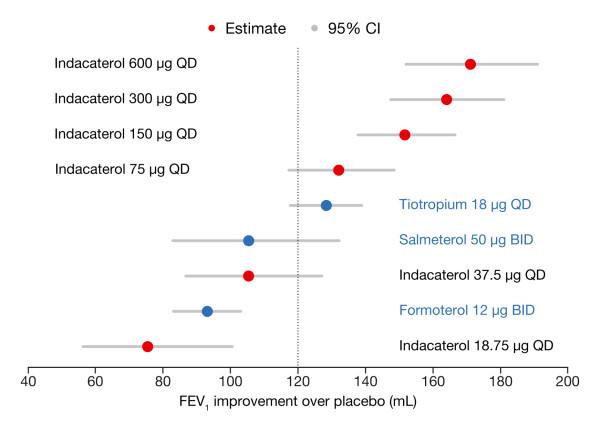
**Ranking of efficacy by dose (study-level analysis)**.

Figure 5 presents the quantification of the dose response, with the response to each indacaterol dose or each comparator ranked by the predicted response. The dots represent the point estimates and the grey lines are the 95% CIs. In this presentation, it is evident that an indacaterol dose of 37.5 μg is less than the MCID and that doses of 75 μg or greater exceed the MCID. However, indacaterol 75 μg overlaps the tiotropium response, whereas indacaterol 150 μg or greater exceeds the tiotropium response. Indacaterol 300 μg is the lowest dose that overlaps the maximum response; indacaterol 150 μg occupies the middle ground between the MCID and the maximum response, and has a response greater than any of the comparators. This analysis suggests that 150 μg is the optimal indacaterol dose.

Since this analysis relied on study-level summaries (LSM), it is possible to assess the predictive performance of these data. This is important, as study-level summaries are often used to support dose-selection decisions for bronchodilators. In figure 3, the light grey shaded area provides the 95% prediction interval for the data, i.e. data from 95% of study visits from trials similar to those used in this programme are expected to fall within this interval of ±60 mL. This is an expression of the difficulty in differentiating doses using conventional approaches for typically sized studies.

### Patient-level analysis

The patient-level analysis, although restricted to the two dose-ranging studies, provided a characterization of dose response that was similar to that obtained in the study-level analysis. The final NLME model used for the patient-level analysis produced a slightly steeper dose-response for the typical COPD patient representative of the population in the two studies. The estimated maximum effect (E_max_) and ED_50 _were respectively 185 mL (95% CI = 163, 210) and 19 μg (95% CI = 10, 36). This translated into indacaterol 18.75 μg providing 49% of the maximum trough FEV_1 _effect, compared with 66% for indacaterol 37.5 μg, 79% for indacaterol 75 μg, 89% for indacaterol 150 μg, 94% for indacaterol 300 μg and 97% for indacaterol 600 μg.

**Figure 6:**
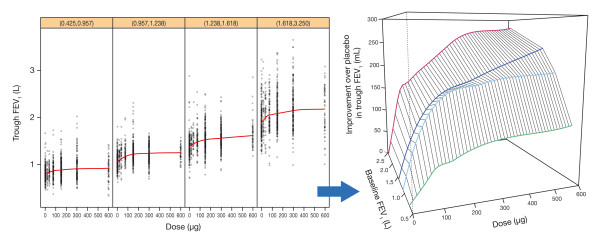
**Left: patient-level dose response data by baseline FEV_1 _category (quartiles) with LOESS curves through the data; Right: zoom-in on smooth curves represented in a three-dimensional manner, after subtracting the placebo effect, to highlight dependency of the response on dose and baseline FEV_1 _(note: the mid-value of the intervals is taken for each baseline FEV_1 _category in the right-hand plot)**.

Unlike the study-level analysis, the patient-level analysis enabled the exploration of patient characteristics that may influence the shape of dose-response. In particular, the covariate search leading to the final NLME model revealed that baseline FEV_1_, which may be considered as a marker of disease severity, was the key covariate. The impact of baseline FEV_1 _on the dose response in the absence of any model-based interpretation is shown in figure 6. The figure shows the individual patient trough FEV_1 _measurements split into quartiles depending on the patients' baseline FEV_1 _values. The LOESS curves in each panel, again intended to highlight the main trends, are also displayed in the right-hand plot, after subtraction of the placebo effect. This gives a visual impression of how the trough FEV_1 _dose response changed with baseline FEV_1_. As baseline FEV_1 _increased, both the steepness and the maximum of the dose response increased. In particular, the lowest quartile, with a baseline FEV_1 _of less than 1 L, had a much flatter dose response.

**Figure 7:**
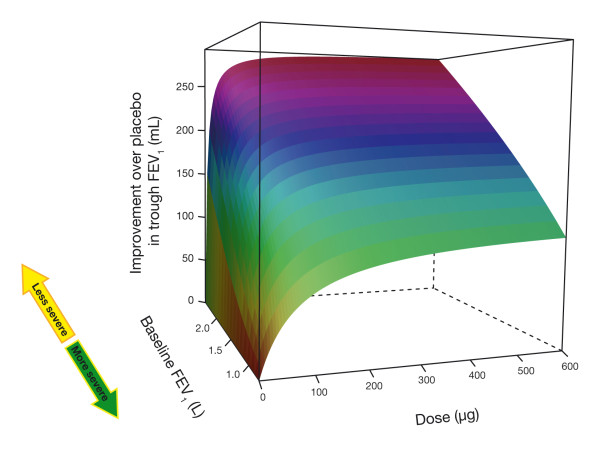
**Three dimensional representation of predicted trough FEV_1 _improvement at steady state for typical COPD patient as a function of dose and baseline value**.

The patient-level model quantifies this overall relationship precisely and demonstrates that both the maximum response (E_max_) and the sensitivity (ED_50_) to a bronchodilator are strongly influenced by the baseline FEV_1_. In other words, as disease severity increases (i.e. baseline FEV_1 _decreases), patients require higher doses to obtain an optimal response. This relationship can be seen in a three dimensional display (figure 7), which highlights the dependency of the trough FEV_1 _response on both dose and baseline FEV_1 _and shows that as baseline FEV_1 _increases, the dose-response curve becomes steeper and reaches a higher maximum level. This analysis suggests that the heterogeneity observed in a typical COPD population may require a more differentiated approach to tailoring therapy to disease status.

**Figure 8:**
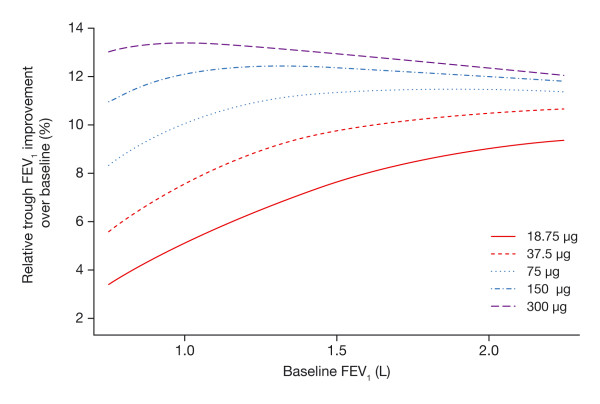
**Impact of baseline FEV_1 _and dose on the improvement in trough FEV_1 _relative to baseline**.

To better understand the relationship between dose and baseline FEV_1 _for patients with differing baseline values, the relative improvement achievable across the dose range was considered. Figure 8 presents the percentage improvement in trough FEV_1 _according to baseline values across the dose range. As baseline FEV_1 _decreases (i.e., severity increases), there is a decrease in the relative improvement across all doses. However, this decrease is strongest for doses of 75 μg or lower. Doses of 150 μg or higher provide sustained bronchodilation that is largely independent of disease severity.

**Figure 9:**
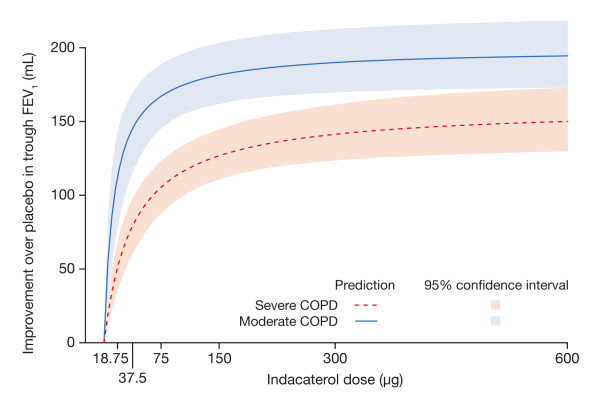
**Prediction of dose response for trough FEV_1 _at steady state in typical patient with moderate or severe COPD according to the predefined GOLD criteria**.

Finally, it is instructive to place the findings of this analysis in the context of the GOLD classification of COPD severity [19]. For this purpose, patients were divided according to the GOLD classification of moderate COPD or better and severe COPD or worse, and the average dose responses for the respective groups were predicted (figure 9). Patients with moderate COPD are predicted to have a steeper dose response with a larger maximum response, whereas patients with severe COPD have a shallower dose response with a lower maximum response. These findings suggest that, for the purpose of effective treatment of COPD, a "one dose fits all" approach may not be most appropriate.

## Discussion

Conventional dose-ranging trials for bronchodilators, such as those used to evaluate tiotropium, salmeterol and formoterol [8,10-12,20], rely on hypothesis testing and use of contrast statistics and do not provide a rigorous basis for identification of the minimally effective, optimal or maximum doses. This is due to the low signal-to-noise ratio inherent in the measurement of FEV_1 _and the poor precision of the conventional methodologies. Simply increasing trial size is not a viable option because the patient numbers required to attain sufficient precision to differentiate active treatments over the dose range would be prohibitively large. To overcome this methodological limitation, alternative approaches were explored using the large indacaterol database to provide a rigorous evaluation of the indacaterol dose response in COPD.

The study-level analysis provided a precise characterization of the dose response using study level data. Data from 11 studies, ranging from 2 to 52 weeks over a dose range from 18.75 to 600 μg were available, including data from 7476 patients and with treatment arm sizes ranging from 65 to 437 patients. Despite the large within- and between-study variability, the analysis was able to meet the requirements of a dose-ranging analysis, namely to precisely differentiate doses over the effective range. Although not shown, a similar pattern was seen in an analysis of peak FEV_1 _(observed peak or area under the curve over 0-4 h post-dose).

Beyond the characterization of the indacaterol dose response itself, the study-level analysis provides unique insights into the precision of the conclusions that may be drawn from typical trials that investigate the efficacy of bronchodilators. Given the typical variability in FEV_1_, it is not possible to precisely determine the metrics such as the MED or differentiate active treatments using pairwise comparisons in typically sized trials. The implication is that the conventional approaches to dose-ranging of bronchodilators cannot easily meet their quantitative objectives. Only through pooling information in a model based approach is it possible to attain the precision necessary to draw robust quantitative conclusions on treatment responses.

While the overall objective of the patient level analysis was also to characterize the dose response, it had the further aim of quantifying the impact of patient characteristics on dose response and ultimately dose selection. The patient level analysis dataset was restricted to the two dose-ranging studies as these were most relevant to the question at hand. Although restriction of the analysis to 2-week data contrasts with the study-level pooled analysis (which pooled data between Week 2 and Month 6), the similar outcomes from the two analyses reinforce the overall conclusions while providing further insights into the impact of patient characteristics on dose response.

The key finding of the patient level analysis was that baseline FEV_1_, as a marker of disease severity, is the most important patient characteristic that influences the dose response. As disease progresses (baseline FEV_1 _decreases) the shape of the dose response changes. However, with doses of 150 μg or greater, the relative response becomes more or less independent of baseline. In other words, doses of 150 μg or greater are required to ensure that patients can achieve optimal benefit. This finding is particularly pertinent to the 25% of the studied COPD population with baseline FEV_1 _less than 1 L. To our knowledge, this analysis is the first to demonstrate and quantify a relationship between COPD severity and dose response.

A number of measures are available for quantifying dyspnoea (e.g. transition dyspnoea index [TDI], the Borg scale and the visual analog scale). TDI is widely used to assess dyspnoea [21] and was the only measure employed consistently across all studies included in our analyses. It measures change from baseline dyspnoea index over time, and comprises three components (functional impairment, magnitude of task and magnitude of effort), each rated from 0 (severe dyspnoea) to 4 (no dyspnoea) [22]. It has been reported that there is a correlation between changes in FEV_1 _and patient-reported outcomes such as TDI [23]. The higher differences from placebo in FEV_1 _with indacaterol doses of 150 μg and higher seen in our analyses would therefore be expected to result in greater improvements in these patient-reported outcomes. In support of this, indacaterol doses of 150 and 300 μg have been shown to result in significantly greater improvements in TDI than placebo in patients with moderate-to-severe COPD, with the 300 μg resulting in numerically (although not statistically) greater improvements than indacaterol 150 μg [2]. This correlation between FEV_1 _and TDI support the concept of identifying the minimum indacaterol doses that provide near maximum bronchodilation so as to optimize the clinical benefit.

It is worth briefly commenting on the presented methods in the context of the original dose selection. Conventional dose-ranging trials rely on hypothesis testing and use contrast statistics to compare dose levels and determine the existence of a dose response. Using placebo corrected means to characterize the dose response and distinguish between doses is not robust if the CIs overlap; for FEV_1 _this is the case even in very large trials. The key difference between the approaches presented in this manuscript and conventional methods is the use of an explicit model, in this case the E_max _model, to pool information across dose levels. It is the pooling of information that provides the greater precision compared to the conventional method, which relies simply on each independent point estimate. In terms of overall efficiency, the patient level analysis of the dose response provides the greatest level of insight for the least number of patients studied. However, a key prerequisite for such an analysis is that data on an adequate dose range is available. In the current analysis, it was necessary to combine two studies to achieve this goal. While this requirement for a wider dose range and larger study population may be considered a drawback of model based methods, it has been suggested this is the price necessary to pay for adequate and robust characterization of the dose response [7].

While the conventional approach originally selected the 150 and 300 μg doses, uncertainty remained about their location on the dose response and, in particular, the efficacy provided by these doses relative to the MCID. The current analyses support the selection of 150 and 300 μg as the lowest doses that ensure optimal response across the spectrum of disease severity, while identifying 75 μg as the MED. The direct clinical benefit of this analysis is that it confirms the selection of doses of indacaterol that provide incremental benefit over other bronchodilators at levels that are at the threshold of the maximum trough response.

In conclusion, thorough analysis of dose response is critical to the successful evaluation of drug treatments in COPD. Model-based approaches such as those described here should allow more informed decisions to be made regarding doses for further evaluation by complementing the results from more classical dose-ranging studies. These comprehensive analyses of the dose response of indacaterol in COPD, showed that 75 μg is the MED of indacaterol and confirms that indacaterol 150 and 300 μg are expected to provide optimal bronchodilation, particularly in patients with severe disease.

## Abbreviations

**(CI)**: confidence interval; **(COPD)**: chronic obstructive pulmonary disease; **(FEV**_**1**_**)**: forced expiratory volume in 1 second; **(LOESS)**: locally weighted scatterplot smoothing; **(LSM)**: least squares mean; **(MCID)**: minimal clinically important difference; **(MED)**: minimum effective dose; **(NLME)**: nonlinear mixed effects; **(TDI)**: transition dyspnoea index

## Competing interests

The authors declare that they have no competing interests.

## Authors' contributions

All authors were involved in the conception and design, or acquisition of data, or analysis and interpretation of data; reviewed each draft of the manuscript and revised it critically for important intellectual content; and approved the final version of the manuscript.

## Supplementary Material

Additional file 1 **Appendix**. Click here for file
