# Supplementary material for: Characterization of the bronchodilatory dose response to indacaterol in patients with chronic obstructive pulmonary disease using model-based approaches
Source: Respir Res. 2011 Apr 26;12(1):54. doi: 10.1186/1465-9921-12-54 (PMC3102616; doi:10.1186/1465-9921-12-54)
Supplement: Additional file 1 — Appendix. [file 1465-9921-12-54-S1.PDF]

Appendix 1

Data sources: covariate adjustments for LSM estimates

Study	Design	Patients	Analysis models
1	Cross-over, 14-day	96	patient + treatment + period + period baseline + daytime + treatment × daytime, with patient included as random effect
2	Parallel-group, 52-week	1732	treatment + baseline + FEV_1 reversibility components + smoking status + country + center (country), with center (country) as a random effect
3	Parallel-group 26-week	2059	treatment + baseline + FEV_1 reversibility components + smoking status + country + center (country), with center(country) as a random effect
4	Crossover, 14-day	68	treatment + period + patient + period baseline with patient included as random effect
5	Parallel-group, 12-week	416	treatment + baseline + FEV_1 reversibility components + smoking status +center, with center as a random effect
6	Parallel-group, 12-week	347	treatment + baseline FEV_1 + FEV_1 reversibility components + smoking status + country + center(country), with center(country) as a random effect
7	Parallel-group, 26-week	563	treatment + baseline FEV_1 + FEV_1 reversibility components + smoking status + theophylline use at screening + country + center(country), with center(country) as a random effect
8	Parallel-group, 26-week	1002	treatment + baseline FEV_1 + FEV_1 reversibility components + smoking status + country + center(country), with center(country) as a random effect
9	Parallel-group, 12-week	323	treatment + baseline FEV_1 + FEV_1 reversibility components + smoking status + ICS use + center, with center as a random effect
10	Parallel-group, 12-week	318	treatment + baseline FEV_1 + FEV_1 reversibility components + smoking status + ICS use + center, with center as a random effect
11	Parallel-group, 2-week	552	treatment + baseline FEV_1 + FEV_1 reversibility components + ICS use + smoking history + center, with center as a random effect

Study-level analysis

We use an E_{max} model,

$$E = \frac{E_{\max} \times dose}{ED_{50} + dose},$$

to characterize the dose-response relationship for indacaterol. The E_{max} parameter describes the maximum possible response, whereas ED_{50} characterizes drug potency and corresponds to the indacaterol dose producing 50% of the maximum effect. *E* is the effect (mean trough response in this case). Since the summary data used in the analysis are contrasts to placebo, the model is constrained to have a null response with placebo (dose=0).

To benchmark indacaterol to other compounds, summary information collected on three comparators (formoterol, salmeterol, tiotropium) was added to the analysis. The complete model equation is:

$$y_{ijk} = FOR_{ij} \times \mu_F + SAL_{ij} \times (\mu_S + \gamma_{Sij}) + TIO_{ij} \times \mu_T \\ + IND_{ij} \times \frac{(E_{\max} + \delta_i + \gamma_{ij}) \times dose_{ij}}{ED_{50} + dose_{ij}} + \varepsilon_{ijk},$$

where:

- y_{ijk} is the trough FEV_1 least-squares mean contrast to placebo at visit k for arm j within study i.
- FOR, SAL, TIO, IND are indicators of whether a record is from formoterol, salmeterol, tiotropium or indacaterol respectively.
- μ_F, μ_S, and μ_T represent the mean effects of formoterol, salmeterol and triotropium respectively.
- γ_{Si} represents the (random) deviation of salmeterol results for study i to the mean effect (μ_S). It is assumed to be randomly distributed with mean 0 and standard deviation $\sigma_{S,A}$. There was only a single and couple of studies available for tiotropium and formoterol, respectively, and the between-arm component was not estimated for those treatments.
- δ_i represents the (random) deviation for study i, and γ_{ij} the (random) deviation for arm j within study i, relative to the typical maximal effect (E_{max}) of indacaterol. Those are assumed to be normally distributed with mean 0 and standard deviation $\sigma_{m,T}$ and $\sigma_{m,A}$, respectively.
- The terms ε_{ijk} are assumed to be normally distributed with mean 0 and standard deviation SE_{ijk}, with SE_{ijk} the estimated standard error originating from the statistical analysis that yielded y_{ijk}. Note the SE_{ijk}'s are considered known (fixed) at this stage of the hierarchy.

To complete the model specification, prior distributions were specified for all model parameters:

- Emax ~ $N(0, 100^2)$ i.e. normal distribution with mean 0 and standard deviation 100.

- $ED_{50} \sim$ unif(0, 600) i.e. a uniform distribution over the dose range 0 to 600 μg.
- $\sigma_{m,T}$, $\sigma_{m,A} \sim$ unif(0, 0.25)
- $\sigma_{S,A} \sim$ exp(45) i.e. exponential distributions with rate parameter equal to 45
- μ_F, μ_S, $\mu_T \sim N(0, 100^2)$

The Bayesian analyses were implemented by using Markov chain Monte Carlo methods with the software WinBUGS v 1.4.3 called from R v 2.10.1, via the library R2WinBUGS tailored to run under the Novartis computing environment.

The iterative algorithm involved monitoring of three Markov chains starting from randomly generated initial values. Each chain was run for a total of 32000 iterations with a thinning rate of 10 and a burn-in of 2000 iterations. This effectively left 3×3000=9000 simulation draws for each parameter in the model. Convergence was assessed by means of the Gelman-Rubin (Rhat) statistic, i.e. the square root of the ratio of the between- to within-chain variances of simulation draws.

The posterior distributions of the structural model parameters were summarized by the mean, SD, and 2.5^{th}, 50th (median), and 97.5^{th} quantiles.

Posterior information was presented graphically with the least-squares summary data overlaid on a plot of the mean dose-response curve on which the 95% confidence and 95% prediction limits were superimposed. The prediction limits were derived by accounting for the between-study variability and uncertainty in least-squares estimates (the median SE value in the dataset being used for the latter).

Patient-level analysis

Efforts were made in the consideration of the base model to incorporate patient level characteristics (covariates) to account for some of the inter-patient differences in dose response. This process was guided by physiological principles such as:

- Baseline FEV_1 is a surrogate measure of disease severity before treatment and is a known, important predictor. It might also be expected that a patient with greater forced expiratory lung volumes at baseline has the potential to show a greater bronchodilatory response when absolute changes in FEV_1 are analysed. That is, baseline FEV_1 should also be an important covariate on Emax.
- Likewise, reversibility of bronchial obstruction following inhalation of short acting beta2 agonists (SABA) should also be an important covariate affecting Emax rather E0, since reversibility is a measure of responsiveness to treatment with beta2 agonists.

The base model equation can be written as follows:

$$y_{ij} = \left[\begin{array}{l} E_0 + E_{0i} + \beta_1 \times base_i \\ + \dfrac{[E_{\max} \times (base_i / mean_{FEV1})^{\beta_2} \times \exp(E_{mi}) + \beta_3 \times (revers_i - mean_{REV})] \times dose_i}{ED_{50} + dose_i} \end{array} \right] \times \varepsilon_{ij},$$

where:

- y_{ij} represents trough FEV_1 measured on patient i at day j.

- E_0 is the intercept fixed effect, and the E_{max} and ED_{50} fixed effects are defined as in the Bayesian mixed-effects model for the meta-analysis.
- *base* represents baseline FEV_1 taken as the average of four FEV_1 measurements available before treatment with indacaterol, that is, two measurements obtained prior to treatment in the reversibility testing sessions and two measurements taken pre-dose on Day 1. Baseline FEV_1 acts on E_0 (additive effect) and E_{max} (multiplicative effect) in the base model.
- $mean_{FEV1}$ denotes the mean value of baseline FEV_1 over the dataset.
- *revers* represents SABA reversibility, that is, the ratio of the difference between post and pre-test FEV_1 values to the pre-test value. SABA reversibility acts on E_{max} (additive effect) in the base model.
- $mean_{REV}$ denotes the mean value of SABA reversibility over the dataset.
- E_{0i} and E_{mi} are random effects to account for inter-patient variation in response, assumed to be independently normally distributed with mean 0 and standard deviation σ_b and σ_m, respectively. Inter-patient variability is additive on E_0 and multiplicative (through a log normal distribution) on E_{max}. Note that correlation between E_{0i} and E_{mi} could not be estimated.
- ε_{ij} denotes the within-patient multiplicative errors, assumed to be independently distributed as log-normal (0, σ) variables.

A transform-both-sides approach was used to deal with the log-normal errors, with the logarithm transformation applied to both the response and the model. In addition to providing some improvement with respect to distributional assumptions of the model (especially in residuals), this has the immediate benefit of ensuring positive values for trough FEV_1, which would not be guaranteed otherwise.

Additional covariates considered in the analysis were: day (trough measurements at Day 14 were uncontrolled, i.e. dosing on the previous day did not take place at the clinic), study, COPD severity (moderate or lower versus severe or worse, based on the classification of severity of COPD defined in GOLD (2007)), use of inhaled corticosteroids, smoking status (ex versus current smoker), gender, age ($<$ versus $\geq$ 65). Race was not considered as more than 88% of the patients were Caucasian in the dataset. A sigmoidal Emax shape was also tested in the model building step. This entails adding a further coefficient (γ) in the E_{max} equation in order to make the model more flexible:

$$E_{\max} \times dose^{\gamma} / (ED_{50}^{\gamma} + dose^{\gamma}) .$$

Starting from the base model, the likelihood ratio test was used to guide model building, the addition of a single parameter being significant at the 5% level when the difference in objective function value (- 2 times log likelihood) between the restricted and full models is greater than 3.84.
